# Antidepressants inhibit P2X_4 _receptor function: a possible involvement in neuropathic pain relief

**DOI:** 10.1186/1744-8069-5-20

**Published:** 2009-04-23

**Authors:** Kenichiro Nagata, Toshiyasu Imai, Tomohiro Yamashita, Makoto Tsuda, Hidetoshi Tozaki-Saitoh, Kazuhide Inoue

**Affiliations:** 1 Department of Molecular and System Pharmacology, Graduate School of Pharmaceutical Sciences, Kyushu University, Fukuoka 812-8582, Japan

## Abstract

**Background:**

Neuropathic pain is characterized by pain hypersensitivity to innocuous stimuli (tactile allodynia) that is nearly always resistant to known treatments such as non-steroidal anti-inflammatory drugs or even opioids. It has been reported that some antidepressants are effective for treating neuropathic pain. However, the underlying molecular mechanisms are not well understood. We have recently demonstrated that blocking P2X_4 _receptors in the spinal cord reverses tactile allodynia after peripheral nerve injury in rats, implying that P2X_4 _receptors are a key molecule in neuropathic pain. We investigated a possible role of antidepressants as inhibitors of P2X_4 _receptors and analysed their analgesic mechanism using an animal model of neuropathic pain.

**Results:**

Antidepressants strongly inhibited ATP-mediated Ca^2+ ^responses in P2X_4 _receptor-expressing 1321N1 cells, which are known to have no endogenous ATP receptors. Paroxetine exhibited the most powerful inhibition of calcium influx via rat and human P2X_4 _receptors, with IC_50 _values of 2.45 μM and 1.87 μM, respectively. Intrathecal administration of paroxetine produced a striking antiallodynic effect in an animal model of neuropathic pain. Co-administration of WAY100635, ketanserin or ondansetron with paroxetine induced no significant change in the antiallodynic effect of paroxetine. Furthermore, the antiallodynic effect of paroxetine was observed even in rats that had received intrathecal pretreatment with 5,7-dihydroxytryptamine, which dramatically depletes spinal 5-hydroxytryptamine.

**Conclusion:**

These results suggest that paroxetine acts as a potent analgesic in the spinal cord via a mechanism independent of its inhibitory effect on serotonin transporters. Powerful inhibition on P2X_4 _receptors may underlie the analgesic effect of paroxetine, and it is possible that some antidepressants clinically used in patients with neuropathic pain show antiallodynic effects, at least in part via their inhibitory effects on P2X_4 _receptors.

## Background

Neuropathic pain is caused by lesions of the central or peripheral nervous system, mainly in patients with diabetes, post-herpetic neuralgia or cancer. Neuropathic pain is especially problematic because of its chronic, severe and intractable pain state, and is characterized by tactile allodynia, which drastically affects the quality of patients' lives. Although a number of patients suffer from neuropathic pain, its pathogenesis is not fully understood. It is widely known that neuropathic pain is nearly always resistant to general analgesics, such as non-steroidal anti-inflammatory drugs or even opioids, but some antidepressants and anticonvulsants have been successful in treating neuropathic pain.

Antidepressants have been used for over 30 years to manage several intractable pain states including chronic headache, low back pain, rheumatoid arthritis and fibromyalgia [1,2]. Accumulated evidence has proved their effectiveness for neuropathic pain states and antidepressants are now considered a mainstay of pharmacological treatment for neuropathic pain, as are anticonvulsants [1]. Tri-cyclic antidepressants (TCAs: amitriptyline, nortriptyline, imipramine, desipramine and clomipramine) have been shown to produce potent analgesic effects in patients with diabetic neuropathy [3-7] and postherpetic neuralgia [8-11]. TCAs achieve analgesic effects at lower doses and with shorter durations of drug exposure than those required to express antidepressive effects [2], indicating putative analgesic mechanisms independent of their antidepressive effect. Among the selective serotonin reuptake inhibitors (SSRIs), it has been shown that fluoxetine and citalopram are less active in treating diabetic neuropathy [12,13]. However, paroxetine (one of SSRIs) has been reported to be effective in patients with diabetic neuropathy [14].

It has been well known that antidepressants induce antidepressive effects via their inhibitory effects on 5-hydroxytryptamine (5-HT) and norepinephrine (NE) transporters in the central nervous system [15]. Monoaminergic neurons descending from the rostral ventral medulla to the spinal cord have been shown to modulate pain transmission, suggesting that inhibition of monoamine transporters may explain the analgesic effects of antidepressants. However, this hypothesis is not fully accepted because antidepressants show non-correlativity between their effectiveness in treating neuropathic pain and their potency of inhibition of monoamine transporters [2,15].

In addition to their inhibitory effects on monoamine transporters, antidepressants have been reported to affect multiple neurotransmitter receptors and ion channels implicated in pain transmission such as NMDA receptors [16,17] and opioid receptors [18]. Recently, it was noted that some antidepressants block several types of sodium channels and calcium channels in recombinant culture [19-22] and neuronal tissue [23]. Although many pharmacological actions of antidepressants have been described, the exact mechanism of action for treating neuropathic pain is not fully understood.

We have recently demonstrated that activating P2X_4 _receptors in activated microglia plays a key role in the pathogenesis of neuropathic pain. Spinal nerve injury induced upregulation of P2X_4 _receptors on activated microglia in the spinal cord and spinal blockade of P2X_4 _receptors induced significant antiallodynic effects [24]. This report strongly suggests that inhibiting P2X_4 _receptors may be a new therapeutic strategy for patients with neuropathic pain, and it is possible that inhibition of P2X_4 _receptors may underlie the analgesic effects of the drugs used to treat patients with neuropathic pain. In the present study, we investigated a possible role of antidepressants as inhibitors of P2X_4 _receptors and analysed their analgesic mechanism using an animal model of neuropathic pain.

## Results

### Antidepressants inhibit rat and human P2X_4 _receptor function

**Figure 1:**
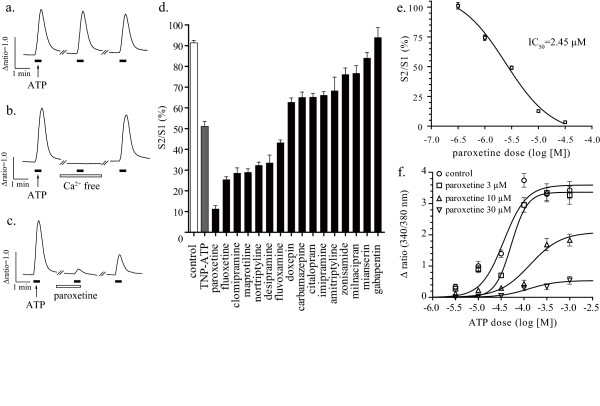
**Effect of antidepressants on the ATP-evoked [Ca^2+^]_i _response via rat P2X_4 _receptors**. Three rounds of ATP stimulation (30 μM, 20 sec) induced [Ca^2+^]_i _response in a reproducible fashion (a). Effect of pretreatment of cells with EGTA (500 μM, 10 min) (b) or with paroxetine (10 μM, 10 min) (c) on [Ca^2+^]_i _response evoked by the second ATP stimulation. Traces indicate 340/380 fura-2 emission ratios averaged from (a) 48 cells, (b) 66 cells and (c) 18 cells obtained from each representative experiment. Effect of pretreatment of cells with TNP-ATP, antidepressants and anticonvulsants (10 μM, 10 min) on the ATP-evoked [Ca^2+^]_i _response via rat P2X_4 _receptors (d). Paroxetine dose-dependently inhibited the [Ca^2+^]_i _response via rat P2X_4 _receptors with an IC_50 _value of 2.45 μM for 30 μM ATP stimulation (e). ATP dose-response curve was generated in the presence of increasing concentrations of paroxetine (f). Data are means ± SEM of at least 10 cells (d, e and f).

**Figure 2:**
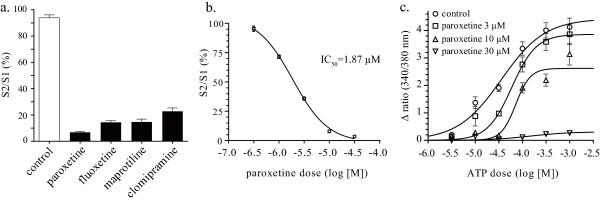
**Effect of antidepressants on ATP-evoked [Ca^2+^]_i _response via human P2X_4 _receptors**. Effect of pretreatment of cells with antidepressants (10 μM, 10 min) on the ATP-evoked [Ca^2+^]_i _response via human P2X_4 _receptors (a). Paroxetine dose-dependently inhibited the ATP-evoked [Ca^2+^]_i _response via human P2X_4 _receptors with an IC_50 _value of 1.87 μM for 30 μM ATP stimulation (b). ATP dose-response curve was generated in the presence of increasing concentrations of paroxetine (c). Data are means ± SEM of at least 3 cells (a, b and c).

**Figure 3:**
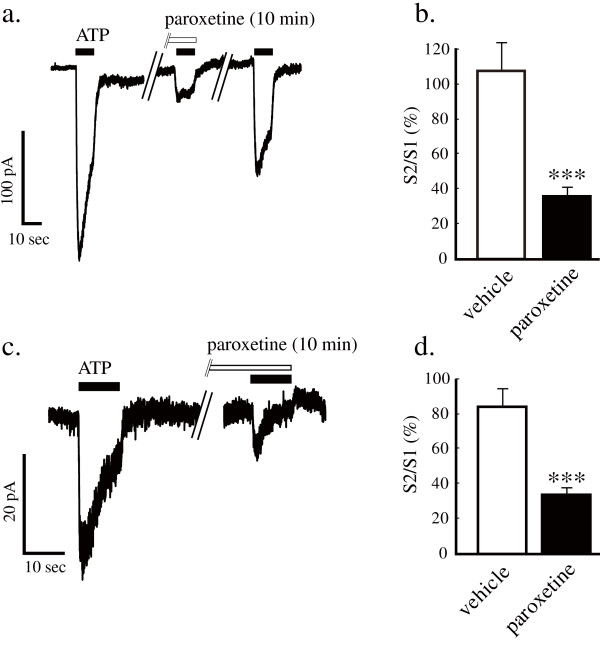
**Effect of antidepressants on ATP-evoked currents in 1321N1 cells expressing rat P2X_4 _receptors and primary microglia**. Paroxetine (10 μM, 10 min) inhibited ATP (30 μM, 10 sec) evoked currents in rat P2X_4 _receptor-expressed 1321N1 cells (a and b) and primary micloglia (c and d) (***p < 0.001; paroxetine vs. vehicle, unpaired t-test). Data are means ± SEM of more than three separate experiments.

To evaluate whether the antidepressants clinically used in patients with neuropathic pain have an influence on P2X_4 _receptors, we used a real-time calcium imaging system to measure intracellular calcium levels in 1321N1 human astrocytoma cells stably expressing rat or human P2X_4 _receptors. Native 1321 N1 cells, which are devoid of ATP receptors, showed no [Ca^2+^]_i _response to ATP stimulation (data not shown). 1321N1 cells stably expressing rat P2X_4 _receptors displayed a reproducible [Ca^2+^]_i _response to ATP stimulation (30 μM, 20 sec) (Figure 1a). The ATP-evoked [Ca^2+^]_i _response disappeared when extracellular calcium was eliminated with EGTA (500 μM) (Figure 1b). Pretreatment of cells with paroxetine (10 μM, 10 min) strongly inhibited the ATP-evoked [Ca^2+^]_i _response via rat P2X_4 _receptors (Figure 1c). Some antidepressants and anticonvulsants (10 μM, 10 min) showed inhibitory effects on the ATP-evoked [Ca^2+^]_i _response via rat P2X_4 _receptors (Figure 1d). Paroxetine dose-dependently inhibited the ATP-evoked [Ca^2+^]_i _response via rat P2X_4 _receptors with an IC_50 _value of 2.45 μM for 30 μM ATP stimulation (Figure 1e). Using 1321N1 cells stably expressing human P2X_4 _receptors, we next determined whether the antidepressants modulate human P2X_4 _receptors. Pretreatment of cells with paroxetine, fluoxetine, maprotiline or clomipramine, which potently inhibit rat P2X_4 _receptors, strongly inhibited the ATP-evoked [Ca^2+^]_i _response via human P2X_4 _receptors (Figure 2a). Paroxetine dose-dependently inhibited the ATP-evoked [Ca^2+^]_i _response via human P2X_4 _receptors with an IC_50 _value of 1.87 μM for 30 μM ATP stimulation (Figure 2b). Both in rat and human P2X_4 _receptor-expressed 1321N1 cells, paroxetine inhibited the maximum response of ATP-evoked [Ca^2+^]_i _increase (Figure 1f and 2c). To elucidate whether paroxetine directly affect recombinant rat P2X_4 _receptors on 1321N1 cells and native P2X_4 _receptors on microglia, the electrophysiological experiments were performed. Pretreatment of cells with paroxetine (10 μM, 10 min) significantly inhibited ATP-evoked currents in rat P2X_4 _receptor-expressed 1321N1 cells (Figure 3a and 3b) and primary micloglia (Figure 3c and 3d).

### Paroxetine produces a significant antiallodynic effect in an animal model of neuropathic pain

**Figure 4:**
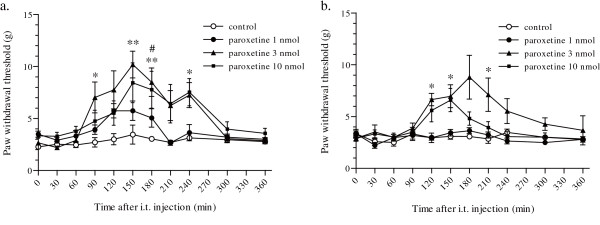
**Effect of intrathecal administration of paroxetine on the decrease in the PWT after nerve injury**. A significant antiallodynic effect was observed following intrathecal administration of paroxetine at doses of 3 nmol and 10 nmol at day 7 (a), and 3 nmol at day 14 (b) after nerve injury (**p < 0.01 and *p < 0.05; paroxetine 3 nmol vs. control, #*p *< 0.05; paroxetine 10 nmol vs. control by a Dunn's multiple comparison test after a Kruskal-Wallis test). Data are means ± SEM of 5–8 rats.

Next, we investigated whether paroxetine, which showed the strongest inhibitory effect on rat and human P2X_4 _receptors, has antiallodynic effect, because we have shown that inhibiting P2X_4 _receptors reversed tactile allodynia in neuropathic rats [24]. A unilateral L5 spinal nerve injury resulted in a marked decrease in the paw withdrawal threshold (PWT) from 15.0 g of pressure (n = 24) before the injury to 3.7 ± 0.2 g (n = 24) at day 7 (Figure 4a) and 3.1 ± 0.8 g (n = 24) at day 14 (Figure 4b) after nerve injury. Intrathecal administration of paroxetine resulted in significant increase in the PWT at doses of 3 nmol (**p < 0.01 and *p < 0.05; Figure 4a) or 10 nmol (#*p *< 0.05; Figure 4a) at day 7 after nerve injury. After the intrathecal administration of 3 nmol paroxetine, the PWT increased gradually, peaking at about 150 min after the injection, and then returned to the pre-injection level. Intrathecal administration of 3 nmol paroxetine also resulted in a significant increase in the PWT at day 14 after nerve injury (*p < 0.05; Figure 4b).

### Fluvoxamine but not citalopram produces an antiallodynic effect in an animal model of neuropathic pain

**Figure 5:**
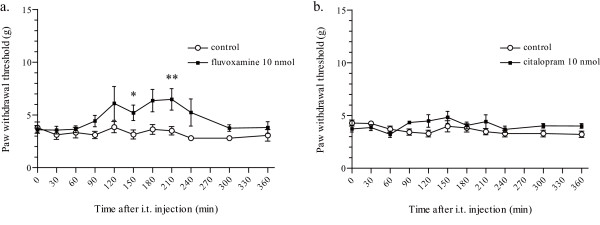
**Effect of intrathecal administration of fluvoxamine and citalopram on the decrease in the PWT after nerve injury**. A significant antiallodynic effect was observed following intrathecal administration of 10 nmol fluvoxamine at day 7 after nerve injury (a). (**p < 0.01 and *p < 0.05; fluvoxamine 10 nmol vs. control by a Mann-Whitney U-test.) No significant change was observed following administration of 10 nmol citalopram (b). (*p *> 0.05 by a Mann-Whitney U-test.) Data are means ± SEM of 5–10 rats.

In order to evaluate whether the potencies of inhibition on P2X_4 _receptors are correlated to the antiallodynic effect, we next investigated other SSRIs, fluvoxamine and citalopram, which have similar pharmacological properties as with paroxetine. Intrathecal administration of 10 nmol fluvoxamine resulted in a moderate increase in the PWT (**p < 0.01 and *p < 0.05; Figure 5a) at day 7 after nerve injury. Intrathecal administration of 10 nmol citalopram had no effect on the PWT at day 7 after nerve injury (*p *> 0.05; Figure 5b).

### Co-administration of 5-HT receptor antagonists did not reverse the antiallodynic effect of paroxetine

**Figure 6:**
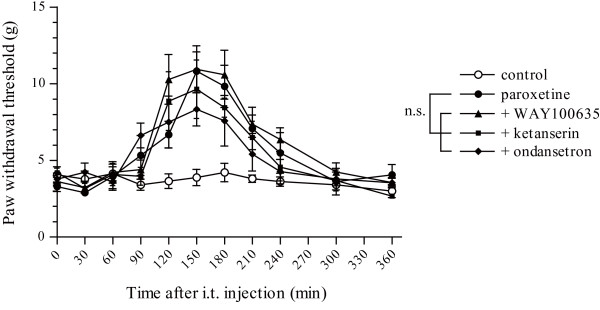
**Effect of intrathecal co-administration of 5-HT receptor blockers with paroxetine on the decrease in the PWT after nerve injury**. No significant change was observed by intrathecal co-administration of 100 nmol WAY100635, 30 nmol ketanserin or 30 nmol ondansetron with 3 nmol paroxetine compared with 3 nmol paroxetine alone. (*p *> 0.05 by a Dunn's multiple comparison test after a Friedman test.) Data are means ± SEM of 5–6 rats.

We next investigated whether 5-HT upregulation induced by inhibition of 5-HT transporters is involved in the antiallodynic effect of paroxetine using antagonists for three types of 5-HT receptors (5-HT_1A_, 5-HT_2A _and 5-HT_3 _receptors), because their abundant expression in the spinal cord and behavioural studies showing pro- or antinociceptive effects have been reported [25-27]. The 5-HT_1A _receptor antagonist WAY100635, the 5-HT_2A _receptor antagonist ketanserin or the 5-HT_3 _receptor antagonist ondansetron were intrathecally co-administered with paroxetine. No significant change in PWT was observed following co-administration of 100 nmol WAY100635, 30 nmol ketanserin or 30 nmol ondansetron with 3 nmol paroxetine compared with 3 nmol paroxetine alone (*p *> 0.05; Figure 6).

### Spinal 5-HT deprivation did not reverse the antiallodynic effect of paroxetine

**Figure 7:**
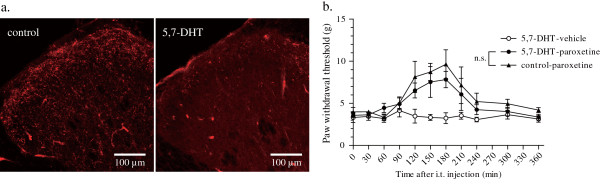
**Effect of spinal 5-HT depletion on the antiallodynic effect of paroxetine**. 5-HT immunoreactivity in the dorsal horn of the spinal cord nine days after intrathecal injection of either saline or 5,7-dihydroxytryptamine (a). A marked reduction in the number of 5-HT immunoreactive fibres was observed in the dorsal horn of the L5 spinal cord after 5,7-DHT treatment compared with the saline-treated group. No significant change in the antiallodynic effect of paroxetine was observed in the 5,7-DHT-treated group compared with the saline-treated group (b). (*p *> 0.05 by a Dunn's multiple comparison test after a Friedman test.) Data are means ± SEM of 5–6 rats.

To further elucidate the interactions between spinal 5-HT system and the antiallodynic effect of paroxetine, we next examined whether pretreatment of cells with 5,7-dihydroxytryptamine (5,7-DHT), which depletes 5-HT in the spinal cord, would affect the antiallodynic effect of paroxetine. Immunohistochemistry revealed that 5-HT immunoreactivity was dramatically reduced throughout the dorsal horn of the spinal cord nine days after 5,7-DHT treatment compared with the saline-treated group (Figure 7a). Double immuno-labelling for P2X_4 _receptors and OX42, a marker for microglia, showed that L5 spinal nerve injury induced upregulation of P2X_4 _receptors on hyperactive microglia at the same level in the 5,7-DHT-treated group as in the saline-treated group, seven days after nerve injury (data not shown). 5,7-DHT-treated rats developed tactile allodynia in the same way as saline-treated rats after nerve injury (Figure 7b). No significant change in the antiallodynic effect of paroxetine was observed in 5,7-DHT-treated rats compared with saline-treated rats (*p *> 0.05; Figure 7b).

## Discussion

We investigated a possible role of antidepressants as analgesics for neuropathic pain based on their inhibitory effects on P2X_4 _receptors. The cDNAs for rat or human P2X_4 _receptors were transfected individually into 1321N1 human astrocytoma cells, which are known to be devoid of endogenous ATP receptor activity and widely used for the analysis of recombinant ATP receptors [28,29]. A lack of responsiveness to ATP stimulation under the calcium-free condition indicates that the ATP-evoked [Ca^2+^]_i _increase in 1321N1 cells was induced by calcium influx from extracellular fluid via P2X_4 _receptors. Recombinant rat or human P2X_4 _receptors expressed in 1321N1 cells showed pharmacological properties similar to those previously described [30,31]. TNP-ATP (10 μM, 10 min), a well known non-selective blocker of rat P2X_4 _receptors, exhibited the same degree of inhibition on ATP-evoked [Ca^2+^]_i _response via rat P2X_4 _receptors as previously reported (IC_50_; 15 μM) [32]. Thus the assay system used here is considered to be appropriate for the screening of P2X_4 _receptor blockers.

For the first time, we found that antidepressants inhibit rat and human P2X_4 _receptor function. Among the drugs used here, paroxetine showed the strongest inhibition of rat and human P2X_4 _receptors, with IC_50 _values of 2.45 μM and 1.87 μM respectively.

In rat and human P2X_4_-expressed 1321N1 cells, the maximum response of ATP-evoked [Ca^2+^]_i _increase was markedly suppressed by paroxetine, suggesting that paroxetine inhibits rat and human P2X_4 _receptors in a non-competitive manner. Using an electrophysiological technique, we found that similar to the results in calcium imaging, the pretreatment of cells with paroxetine strongly inhibited the ATP-induced currents on rat P2X_4 _receptor-expressed 1321N1 cells. Therefore, it is proposed that paroxetine directly inhibits P2X_4 _receptors. Furthermore, paroxetine strongly inhibited the ATP-induced currents on primary cultured microglial cells. We have previously shown that an exposure of such concentration of ATP to primary microglia selectively activates P2X_4 _receptors [33]. These findings indicate that paroxetine inhibits native P2X_4 _receptors expressed in microglia.

In general, typical serum concentrations of antidepressants range from about 100 to 1000 nM [15]. Antidepressants tend to accumulate in tissues because of their lipophilic nature [34], so in the central nervous system they may reach the effective range for inhibition of P2X_4 _receptors observed in this experiment. Antidepressants modulate many kinds of ion channels at a wide range of concentrations (0.1 to 1000 μM) *in vitro *[20,35,36], but only the effects observed near the serum concentration are considered to have an influence *in vivo*. Sometimes, the analgesic effect of antidepressants is explained by their inhibitory effects on voltage-dependent sodium channels and calcium channels, that are observed at relatively low concentrations (0.1 to 10 μM) *in vitro *[19]. For example, paroxetine showed inhibitory effects on hNav1.3 (effective range; >2 μM) [21] and hNav1.7 (Ki = 1.45 μM) [20], at concentrations very close to that needed to affect P2X_4 _receptors in this experiment. These findings indicate that antidepressants may have some influence on spinal P2X_4 _receptors in patients with neuropathic pain.

Intrathecal administration of paroxetine showed a potent antiallodynic effect at 7 days and 14 days after nerve injury. We have previously shown that intrathecal administration of TNP-ATP induces significant antiallodynic effects at higher doses (10 or 30 nmol) [24] than paroxetine, indicating that there is a correlation between the dose needed to express the antiallodynic effect *in vivo *and the potency of inhibition of P2X_4 _receptors *in vitro*. The antiallodynic effect of paroxetine was greater at day 7 than day 14 after nerve injury, which is a common feature with the antiallodynic action of intrathecally administered TNP-ATP. In several time points both at day 7 and day 14, paroxetine was more effective at 3 nmol than 10 nmol. It has been reported that paroxetine increases [Ca^2+^]_i _level at high concentrations greater than 50 μM and induces apoptosis in MG63 cells [37]. The weak antiallodynic effect of paroxetine 10 nmol may be due to its cell toxicity.

We also found that fluvoxamine produced a much weaker antiallodynic effect than paroxetine, and citalopram produced no antiallodynic effect, although these SSRIs (paroxetine, fluvoxamine and citalopram) have similar inhibitory action on 5-HT transporters. Citalopram has been reported to be less effective than paroxetine in patients of diabetic neuropathy [14,38]. Interestingly, we found that citalopram (10 μM, 10 min) had no effect on ATP-evoked [Ca^2+^]_i _response mediated by human P2X_4 _receptors (additional file 1: Effect of citalopram on ATP-evoked [Ca^2+^]_i _response via human P2X_4 _receptors). These results indicate that the difference in the potency of inhibition on P2X_4 _receptors may explain the difference in the clinical effectiveness of antidepressants in patients of neuropathy.

It has been well known that microglia express P2X_7 _receptors as well as P2X_4 _receptors [39]. We observed that paroxetine (10 μM, 10 min) inhibited BzATP (100 μM, 20 sec) induced [Ca^2+^]_i _response of P2X_7 _receptor-expressed 1321N1 cells (additional file 2: Effect of paroxetine on BzATP-evoked [Ca^2+^]_i _response via rat P2X_7 _receptors). Therefore, it is conceivable that intrathecally administered paroxetine may also inhibit P2X_7 _receptors in the spinal cord. However, we have previously shown that PPADS, a non-selective antagonist for P2X receptors including P2X_7_, has no effect on mechanical allodynia in neuropathic pain model [24]. Therefore, these results suggest that subtypes of P2X receptors sensitive to PPADS are not involved in the antiallodynic effect of paroxetine under our experimental conditions.

It has been widely accepted that serotonergic neurons descending from the rostral ventral medulla into the spinal cord participate in endogenous antinociceptive mechanisms. Activation of this descending inhibitory pathway or intrathecal administration of 5-HT induced analgesia in several behavioural tests [40-42]. The main pharmacological action of paroxetine is an inhibition of 5-HT transporters, which induces upregulation of 5-HT [15]. Thus we next investigated whether the spinal 5-HT system is involved in the antiallodynic effect of paroxetine. We focused on three subtypes of 5-HT receptors (5-HT_1A_, 5-HT_2A _and 5-HT_3 _receptors) because of their abundant expression in the spinal cord [25-27] and behavioural studies showing pro- or antinociceptive effects induced by intrathecal administration of selective drugs for them. In neuropathic rats, systemic administration of F 13640, a 5-HT_1A _receptor agonist, attenuated tactile allodynia [43] and intrathecal administration of α-methyl-5-HT maleate, a 5-HT_2A _receptor agonist, attenuated thermal hyperalgesia, which was abolished by pretreatment with ketanserin [44]. In the spinal cord injury model, a pro-nociceptive effect has been observed following intrathecal administration of m-chlorophenylbiguanide, a 5-HT_3 _receptor agonist [45]. These reports indicate pain modulation by the spinal 5-HT system in neuropathic rats, but we observed no significant change in the antiallodynic effect of paroxetine following co-administration with 5-HT receptor blockers.

In neuropathic rats, it has been reported that spinal administration of 5-HT produced only a weak analgesia and needed a 100- to 1000-fold higher dose than that required to achieve the antinociceptive effect in normal rats [40,46]. This indicates some physiological changes in the serotonergic system in the spinal cord of neuropathic rats, leading to less analgesia induced by spinal 5-HT administration. In this report, the 5-HT upregulation induced by spinal-administered paroxetine may not be involved in the antiallodynic effect in the same way as in the previous report.

It has been well established that intrathecal administration of 5,7-DHT depletes spinal 5-HT content [45,47], and we observed a significant reduction of spinal 5-HT immunoreactivity at day 9 after intrathecal administration of 5,7-DHT. Spinal 5-HT depletion induced no significant changes in the degrees of tactile allodynia and immunoreactivity for OX42 and P2X_4 _receptors at day 7 after nerve injury. No significant change in the antiallodynic effect of paroxetine in 5,7-DHT-treated rats supports a putative antiallodynic mechanism independent of the spinal 5-HT system.

## Conclusion

In this study, we found that some antidepressants and anticonvulsants clinically used in patients with neuropathic pain have inhibitory effects on rat and human P2X_4 _receptor function. Among the drugs used, paroxetine showed the strongest inhibition on rat and human P2X_4 _receptor function. Intrathecal administration of paroxetine and fluvoxamine, but not citalopram, resulted in an antiallodynic effect in an animal model of neuropathic pain, which correlates the potency of inhibition of rat P2X_4 _receptors. Co-administration of 5-HT receptor antagonists (WAY100635, ketanserin or ondansetron) and spinal 5-HT depletion did not reverse the antiallodynic effect of paroxetine, which indicates an antiallodynic mechanism independent of the spinal 5-HT system. Powerful inhibition of P2X_4 _receptors may be responsible for the analgesic effect of paroxetine and it is possible that some antidepressants clinically used in patients with neuropathic pain produce antiallodynic effects mediated at least in part via their inhibitory effect on P2X_4 _receptors.

### Methods

#### Culturing 1321N1 cells

The cDNAs encoding rat and human P2X_4 _receptors [provided by Prof. Susumu Seino (Kobe University Graduate School of Medicine, Hyogo) and Prof. Joji Ando (The University of Tokyo, Japan), respectively] incorporated into pcDNA3.1+ (Clontech Laboratories, Inc., Mountain. View, CA) [48] were introduced into 1321N1 human astrocytoma cells (a gift from Dr. Michael W. Salter, University of Toronto, Toronto, Canada) using FuGENE6 transfection reagent (Roche Applied Sciences, Basel, Switzerland). 1321N1 cells stably expressing P2X_4 _receptors were maintained in Dulbecco's modified Eagle's medium supplemented with 10% fetal bovine serum in a humidified atmosphere of 95% air and 5% CO_2 _at 37°C and split 1/6 every three days. For the measurement of [Ca^2+^]_i_, the cells were plated onto poly-L-lysine-coated glass coverslips, placed in silicon rubber walls (Flexiperm, Greiner Bio-One GmbH, Frickenhausen, Germany) and maintained for about 48 hr.

### Culturing primary microglia

Primary cultured microglia were prepared according to the method described previously [24]. In brief, the mixed glial culture was prepared from brain of neonatal Wistar rats (Kyudo, Saga, Japan) and maintained for 9–15 days in DMEM with 10% fetal bovine serum. Microglia were obtained as floating cells over the mixed glial culture. The floating cells were collected by gentle shaking and transferred to culture dishes and then the microglia were cultured for 1–6 h and used for whole-cell patch clamp. The cultures were of >99% purity, determining by immunostaining for OX-42 and Iba1 [33].

### Measurement of [Ca^2+^]_i _in single cells

[Ca^2+^]_i _in single cells was monitored by a fura-2 ratio imaging system. The cells were incubated with 2.5 μM fura-2AM (Wako Pure Chemical Industries, Ltd., Osaka, Japan) for 45 min in a balanced salt solution (BSS; composition in mM: NaCl 150, KCl 5, CaCl_2 _1.8, MgCl_2 _1.2, D-glucose 10 and HEPES 25; pH 7.4) at room temperature. Then, the cells were washed with BSS and mounted on an inverted fluorescence microscope (ECLIPSE TE2000-U: Nikon, Tokyo, Japan) equipped with a Xenon-lamp (Xe75W; Nikon) and band-pass filters of 340 nm and 380 nm. The emission fluorescence was measured at 510 nm. Image data were detected with Aquacosmos (Hamamatsu Photonics, Hamamatsu, Japan), and [Ca^2+^]_i _was expressed as the ratio of the fluorescence intensities at 340 nm and 380 nm. Applying 30 μM ATP for 20 sec to the 1321N1 human astrocytoma cells expressing rat or human P2X_4 _receptors, a first [Ca^2+^]_i _response (S1) was measured. Drugs were added to the cells for 10 min, and [Ca^2+^]_i _response (S2) was measured by a second ATP application. Inhibitory effects of the drugs were evaluated by the S2/S1 ratio. After washing out the drugs by BSS, we confirmed recovery of [Ca^2+^]_i _response by a third ATP stimulation.

### Whole-cell patch clamp

Whole-cell currents were recorded at a holding potential of – 60 mV with Patch clamp L/M-EPC7 (List Medical-Electronic). The cells were placed in a recording chamber and continuously superfused at room temperature (22 – 24°C) in an extracellular solution composed of the following: 140 mM NaCl; 5 mM KCl; 2.5 mM CaCl_2_; 1 mM MgCl_2_; 10 mM HEPES; and 10 mM D-glucose, and the pH was adjusted to 7.4 with NaOH. Patch pipettes were filled with buffer containing: 130 mM KCl; 1 mM CaCl_2_; 2 mM MgCl_2_; 10 mM HEPES and 10 mM EGTA, and the pH was adjusted to 7.2 with CsOH. All experimental parameters were controlled using Clampex software (version 9, Molecular Devices) and analyzed with Clampfit (version 9, Molecular Devices). All solutions were applied using custom made Y-tube apparatus. Applying 30 μM ATP for 10 sec to the cells, a first response (S1) was measured. Drugs were added to the cells for 10 min, and a second response (S2) was measured by an ATP application. Inhibitory effects of the drugs were evaluated by the S2/S1 ratio. After washing out the drugs by external solution, we confirmed recovery of response by a third ATP stimulation.

### Animals

Male Wistar rats weighing 250–270 g were used in this study. Rats were housed at a temperature of 22 ± 1°C with a 12-h light/dark cycle (light on 8:30 to 20:30) and were fed food and water ad libitum. All of the animals used in the present study were treated in accordance with the guidelines of Kyushu University.

### Neuropathic pain model

We used the spinal nerve injury model [49] with some modifications. A unilateral L5 spinal nerve of rats was tightly ligated and cut just distal to the ligature under isoflurane (2.5%) anesthesia. To assess tactile allodynia, calibrated von Frey filaments (0.4–15.1 g, Stoelting Co., Wood Dale, IL) were applied to the plantar surface of the hindpaw from below the mesh floor. The 50% paw withdrawal threshold was determined by the up-down method [50,51]. Drugs were intrathecally administered to rats 7 days or 14 days after nerve injury and tactile allodynia was measured for 6 hr.

### Intrathecal drug administration

Surgery to place an indwelling catheter was conducted about 5–7 days before spinal nerve ligation. Under isoflurane (2.5%) anesthesia, rats were implanted with catheters for intrathecal injection according to a method described previously [52]. A polyethylene tube was inserted through the atlanto-occipital membrane to the lumbar enlargement (close to the L4-L5 segments) and externalized through the skin. Rats were injected intrathecally with drugs using a 25-μl Hamilton syringe with 28-gauge needle.

### 5,7-DHT administration

Rats were pretreated with desipramine hydrochloride (20 mg/kg, dissolved in 5% DMSO in saline, i.p., Sigma-Aldrich, Saint Louis, MO) to prevent uptake of the 5,7-DHT into noradrenergic neurons. After 45 min, rats received intrathecal injection of either saline or 5,7-DHT (100 μg, dissolved in 1% ascorbic acid in saline, Sigma-Aldrich) in a volume of 20 μl followed by 10 μl saline flush. This dose of 5,7-DHT has been reported to be sufficient to deplete endogenous spinal 5-HT [45,47]. Spinal nerve ligation was conducted 2 days after 5,7-DHT administration.

### Immunohistochemistry

Seven days after spinal nerve ligation, vehicle or 5,7-DHT treated rats were deeply anesthetized with pentobarbital (100 mg/kg, i.p.) and perfused transcardially with 100 ml of phosphate-buffered saline (PBS, composition in mM: NaCl 137, KCl 2.7, KH_2_PO_4 _1.5, NaH_2_PO_4 _8.1; pH 7.4), followed by 250 ml of ice-cold 4% paraformaldehyde. The fifth lumbar (L5) segments of the spinal cord sections was removed and postfixed at 4°C for 5 hr and then transferred to 30% sucrose/PBS for 24 hr. Transverse L5 spinal cord sections (30 μm) were incubated for 2 hr at room temperature in a blocking solution (3% normal goat serum) and then incubated for 48 hr at 4°C with rat anti-serotonin monoclonal antibody (1:100, Millipore Corporation, Billerica, MA), mouse anti-OX42 antibody (1:1000, Chemicon, Temecula, CA) or rabbit anti-P2X_4 _receptor antibody (1:1000, Alomone Labs, Jerusalem, Israel). Following incubation, tissue sections were washed and incubated for 3 hr at room temperature in the secondary antibody solution (goat anti-rat IgG-conjugated Alexa Fluor 546, goat anti-mouse IgG-conjugated Alexa Fluor 546 or goat anti-rabbit IgG-conjugated Alexa Fluor 488, 1:1000, Molecular Probes, Eugene, OR). The spinal cord sections were analysed using an LSM confocal imaging system (Carl Zeiss Japan, Tokyo, Japan).

### Drugs

For *in vitro *experiments, adenosine 5'-triphosphate disodium salt (ATP), amitriptyline hydrochloride, citalopram hydrochloride, clomipramine, desipramine hydrochloride, doxepin hydrochloride, fluvoxamine maleate, imipramine hydrochloride, maprotiline, mianserin hydrochloride, milnacipran hydrochloride, nortriptyline hydrochloride, zonisamide sodium salt and TNP-ATP were purchased from Sigma-Aldrich and dissolved in BSS. Gabapentin (Toronto research chemicals Inc., North York, Ontario, Canada) and fluoxetine HCl (Biomol, Philadelphia, PA) were dissolved in BSS. Carbamazepine (Sigma-Aldrich) and paroxetine hydrochloride (Toronto Research Chemicals Inc.) were dissolved in 0.1% dimethyl sulfoxide (DMSO) in BSS. For *in vivo *experiments, paroxetine, fluvoxamine and citalopram were dissolved in 5% DMSO in PBS. WAY100635 (Sigma-Aldrich), ketanserin (Sigma-Aldrich) and ondansetron (Sigma-Aldrich) were dissolved in 5% DMSO in saline for co-administration with paroxetine.

### Statistical analysis

Differences between groups were analyzed using an unpaired t-test, a Friedman test with a Dunn's multiple comparison post-hoc test, a Kruskal-Wallis test with a Dunn's multiple comparison post hoc-test or a Mann-Whitney U-test. A *p *value < 0.05 was considered to be statistically significant.

## Abbreviations

TCAs: tri-cyclic antidepressants; SSRIs: selective serotonin reuptake inhibitors; 5-HT: 5-hydroxytryptamine; NE: norepinephrine; 5,7-DHT: 5,7-dihydroxytryptamine; BSS: balanced salt solution; PBS: phosphate-buffered saline; ATP: adenosine 5'-triphosphate disodium salt; DMSO: dimethyl sulfoxide.

## Competing interests

The authors declare that they have no competing interests.

## Authors' contributions

KN, TI, MT and KI were responsible for experimental design. KN and TY participated in calcium imaging. HT participated in electrophysiology. KN and TI participated in animal surgery and behavioural experiments. KN participated in immunohistochemical experiments. KN, TI, MT and KI participated in manuscript writing. All authors read and approved the final manuscript.

## Supplementary Material

Additional File 1 **Effect of citalopram on ATP-evoked [Ca^2+^]_i _response via human P2X_4 _receptors**. Effect of pretreatment of cells with citalopram (10 μM, 10 min) on the ATP-evoked [Ca^2+^]_i _response via human P2X_4 _receptors. Citalopram has no effect on the ATP-evoked [Ca^2+^]_i _response via human P2X_4 _receptors. Data are means ± SEM of 164–181 cells. Click here for file

Additional File 2 **Effect of paroxetine on BzATP-evoked [Ca^2+^]_i _response via rat P2X_7 _receptors**. Paroxetine (10 μM, 10 min) significantly inhibited the BzATP (100 μM, 20 sec) induced [Ca^2+^]_i _response in rat P2X_7_-expressed 1321N1 cells (***p < 0.001 by unpaired t-test). Data are means ± SEM of 95–113 cells. Click here for file
