# Supplementary figures and images for: Antidepressants inhibit P2X4 receptor function: a possible involvement in neuropathic pain relief
Source: Mol Pain. 2009 Apr 23;5:20. doi: 10.1186/1744-8069-5-20 (PMC2680826; doi:10.1186/1744-8069-5-20)

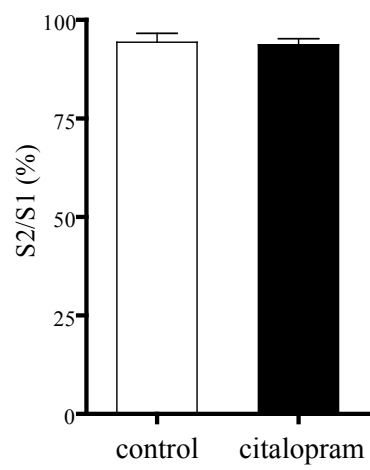

Supplement: Additional File 1 — Effect of citalopram on ATP-evoked [Ca2+]i response via human P2X4 receptors. Effect of pretreatment of cells with citalopram (10 μM, 10 min) on the ATP-evoked [Ca2+]i response via human P2X4 receptors. Citalopram has no effect on the ATP-evoked [Ca2+]i response via human P2X4 receptors. Data are means ± SEM of 164–181 cells. [file 1744-8069-5-20-S1.pdf]

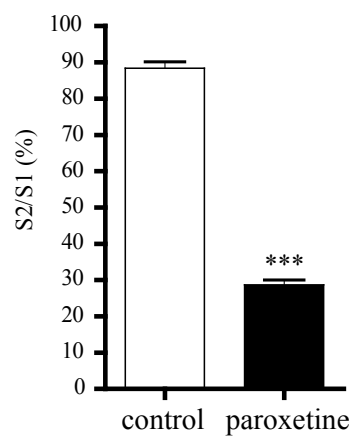

Supplement: Additional File 2 — Effect of paroxetine on BzATP-evoked [Ca2+]i response via rat P2X7 receptors. Paroxetine (10 μM, 10 min) significantly inhibited the BzATP (100 μM, 20 sec) induced [Ca2+]i response in rat P2X7-expressed 1321N1 cells (***p < 0.001 by unpaired t-test). Data are means ± SEM of 95–113 cells. [file 1744-8069-5-20-S2.pdf]
